# The Application of Optical Coherence Tomography to Image Subsurface Tissue Structure of Antarctic Krill *Euphausia superba*

**DOI:** 10.1371/journal.pone.0110367

**Published:** 2014-10-13

**Authors:** Nicola Bellini, Martin J. Cox, Danielle J. Harper, Sebastian R. Stott, Praveen C. Ashok, Kishan Dholakia, So Kawaguchi, Robert King, Tammy Horton, Christian T. A. Brown

**Affiliations:** 1 SUPA, School of Physics and Astronomy, University of St Andrews, North Haugh, St Andrews, Fife, United Kingdom; 2 Australian Antarctic Division, Kingston, Tasmania, Australia; 3 National Oceanography Centre, University of Southampton Waterfront Campus, Southampton, United Kingdom; German Cancer Research Center, Germany

## Abstract

Many small open ocean animals, such as Antarctic krill, are an important part of marine ecosystems. To discover what will happen to animals such as krill in a changing ocean, experiments are run in aquaria where conditions can be controlled to simulate water characteristics predicted to occur in the future. The response of individual animals to changing water conditions can be hard to observe, and with current observation techniques it is very difficult to follow the progress of an individual animal through its life. Optical coherence tomography (OCT) is an optical imaging technique that allows images at high resolution to be obtained from depths up to a few millimeters inside biological specimens. It is compatible with *in vivo* imaging and can be used repeatedly on the same specimens. In this work, we show how OCT may be applied to *post mortem* krill samples and how important physiological data such as shell thickness and estimates of organ volume can be obtained. Using OCT we find an average value for the thickness of krill exoskeleton to be (30±4) µm along a 1 cm length of the animal body. We also show that the technique may be used to provide detailed imagery of the internal structure of a pleopod joint and provide an estimate for the heart volume of (0.73±0.03) mm^3^.

## Introduction

Marine ecosystems, particularly high-latitude systems, are vulnerable to the effects of ocean acidification [1]. Aquaria-based studies have revealed Antarctic krill *Euphausia superba* [2] (hereafter krill) to be vulnerable to elevated pH throughout their lifecycle, with reduced hatching success [3], increased physiological costs [4], and possible disruption to growth and moulting during later life-cycle stages [5]. In addition to direct observation of hatching success and growth rates, morphological measurements of krill are important when determining condition, exoskeleton thickness, and reproductive state. Here we demonstrate the suitability of optical coherence tomography (OCT), an optical non-invasive imaging technique, to the observation of small pelagic animal morphology, using krill as an example species.

Due to their ecological importance, krill were selected as an example species to be studied with OCT. Krill are the dominant consumer in the Southern Ocean [6] and are an important prey item for many species of fish, penguin, seal and whale [7], [8] forming a vital component in the Antarctic foodweb [9] as well as being a commercial fishery resource [10]. In addition to their ecological importance, the translucent tissue composition of krill makes them particularly amenable to OCT allowing the identification and observation of interior structures including some of those shown in the detailed diagram presented in figure 40 of the work of Mc^c^Laughlin [11].

OCT is an optical measurement and imaging technique initially developed in the early 1990’s [12]. In OCT, light is directed onto the surface of an object and the backscattered signal is measured. Using interferometric techniques the reflections from various structures beneath the surface of the object can be retrieved. From these reflections, the profile of the sub-surface structures can be imaged. OCT is an optical analogue to ultrasound measurement but provides much higher spatial resolution images (typically 10 µm versus mm for ultrasound) at the expense of a greatly reduced penetration depth (typically 2 mm versus cm for ultrasound). OCT has grown into a widely used technology in the physical and medical sciences that finds applications from human retinal monitoring to the detection and classification of sub-surface tumours [13], [14].

Using OCT provides a unique capability to characterise near-surface tissue features with relatively high resolution, enabling the formation of highly-informative images of internal structures. In addition to qualitative images, OCT can also provide quantitative measurements since all OCT images are spatially referenced so distances can be measured from surface or sub-surface tissues of interest.

There are two key attributes of OCT that make it an important observation tool for ecological studies:

1. OCT is a non-invasive, non-toxic imaging technique that is well suited to the measurement of biological tissues without causing damage to the sample (due to the very low incident optical power). Crucially, this means it may be applied *in vivo* as well as on the frozen sample types discussed in this report. Many of the current laboratory-based observation techniques require the destruction of individual specimens in order to obtain data. This prevents repeated measurements upon individuals, making it impossible to observe an individual specimen through a life cycle stage, and may increase the cost of an experiment.
2. A typical OCT set-up provides high-resolution locally spatial-referenced images and provides axial resolution of ∼10 µm to depths of a few mm depending on the properties of tissue sample and the wavelength and bandwidth of the optical source used. The transverse resolution of the images is dictated by the optical set-up and is typically comparable to the axial resolution. Local spatial referencing allows distances to be measured, 3D images created, and volumes and areas calculated.

## Materials and Methods

### Krill specimens

Krill specimens were a mixture of wild-caught and aquaria reared specimens, flash frozen in liquid nitrogen, then stored frozen. Live wild krill were caught in the Southern Ocean under permits issued by the Australian Department of Environment and Heritage under the Environment Protection and Biodiversity Conservation Act 1999, permit WT2005-8619 and under the Antarctic Marine Living Resources Conservation Act 1981, permit AMLR 05-06_2655. Overall we used and processed 50 krill samples.

### OCT experimental set-up

**Figure 1:**
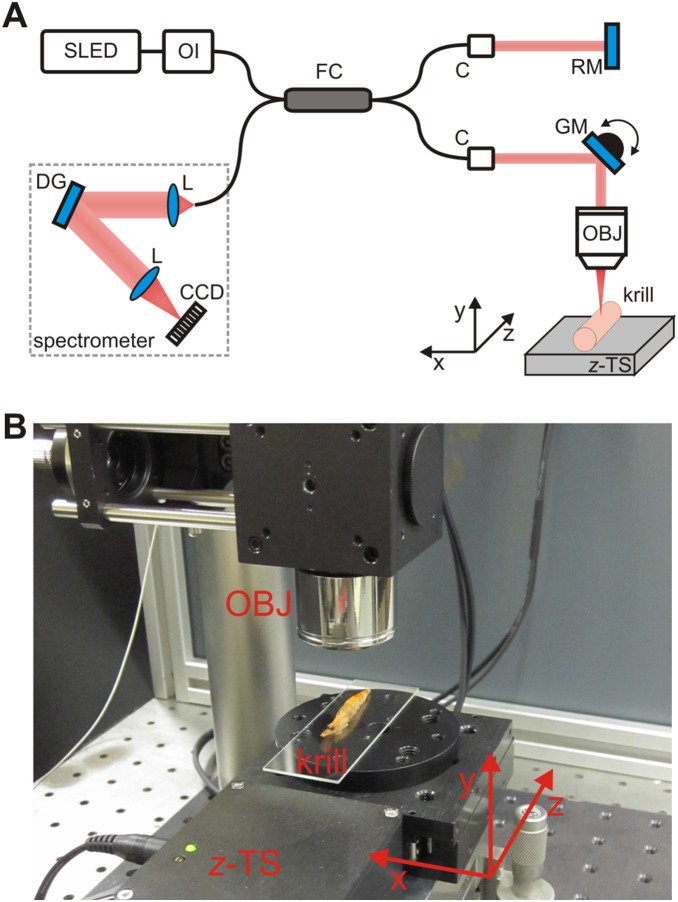
Layout of OCT set-up. (A) Scheme of the OCT set-up employed for experiments. (B) Picture of a krill sample under OCT scanning. The following abbreviations are used: SLED superluminescent light emitting diode, OI optical isolator, FC fiber coupler, C fiber collimator, RM reference mirror, GM galvanometric mirror, OBJ focusing objective, *z*-TS computer-controlled translation stage for *z*-axis motion, L lens, DG diffraction grating, CCD 1D charge-coupled device for signal detection.

The home-developed OCT set-up used is illustrated in Fig. 1 and is described in detail elsewhere [15]. Briefly, the OCT system is a standard fiber-based Fourier-domain OCT set-up. The superluminescent light emitting diode (SLED) used as a light source for the experiments operates in continuous wave (CW) at a central wavelength of 840 nm, a spectral region that offers good transparency and low scattering loss in most biological tissues. The incident optical power on the sample was measured to be about 2 mW. The signal detection is based on a high-sensitivity 1D spectrometer built in house.

Such an OCT system provides a cross-sectional *xy* image of a stationary sample, where *x* is the horizontal lateral position and *y* is the vertical depth direction into the sample. The OCT system provides a horizontal image size of 5 mm along *x*, and a maximum vertical penetration depth along *y* of 1.7 mm in air. In order to scale the images and provide an estimate for morphology dimensions, the vertical axis *y* has to be corrected for the refractive index of the tissue. We have calibrated our system using commercially available frozen raw prawn and shrimp samples from which we have derived a refractive index of 1.45±0.05 for the krill body tissue (see File S1) which we used to rescale all processed OCT images. In practice, this index is expected to vary due to natural heterogeneities in the optical density of krill; this variation will translate to an error of about 3.4% in all vertical measurements. Using this refractive index, the penetration depth along the vertical direction *y* in krill was about 1.2 mm. With the same procedure and commercially available samples, we measured the refractive index of the exoskeleton to be 1.56±0.05 (see File S1); we used this value for exoskeleton thickness evaluation in krill. The resolution in the OCT image depends on the properties of the optical set-up: a 5× telecentric objective was used as the focusing lens in the sample arm, resulting in a lateral resolution of 17 µm (*x*-axis); the SLED had a bandwidth of 50 nm, ensuring an axial resolution of about 6 µm (*y*-axis). Since the SLED operates in CW, the optical intensity impinging on the sample is less than 900 W/cm^2^, a value far below the damage level in tissue [14].

The OCT system could be operated in two modes. In the first one, single line scans may be taken of a particular region of the specimen. Operating in this mode, a single *xy* cross-sectional OCT image was acquired and saved in about 1 s, potentially allowing this to be applied to live animals. In the second mode of operation, which allowed the reconstruction of a 3D image of a full krill specimen, an external computer-controlled linear translation stage (Fig. 1; component *z*-TS) with sub-micrometric precision was used and synchronized with the OCT image acquisition system. This translation stage performed the movement of the sample along the lateral direction *z* in steps of 15 µm. In this way, all the 3D information was recorded in a stack of consecutive OCT *xy*-images along the full body of the sample over a sampling period of several 10 s of minutes.

### Image analysis

Qualitative information can be obtained immediately by looking at a single cross-sectional OCT image. This is already quite rich in sub-surface feature information not visible by eye. Also, on a single cross-sectional *xy* image, the distance between any two points can be easily measured once the image has been vertically rescaled as discussed previously.

As an example of the powerful OCT capabilities, exoskeleton thickness along the entire body length was determined. For this purpose, a MATLAB code was specifically written for automatic exoskeleton recognition and measurement. Firstly, in a single *xy* cross-sectional OCT image, we extracted the image intensity profile along the vertical *y*-axis for each lateral position *x*; in each intensity profile, the exoskeleton position was detected as the peak position since the exoskeleton presents the highest contrast in the image. Then, the width of the peak at ¾ of its maximum was measured to discard background noise and intensity oscillations; considering the peak of Gaussian-like shape, the full-width at half-maximum (FWHM) was retrieved and assumed as the thickness for such an *x* position. In order to have a more consistent thickness value, the measurement was repeated over a certain range along *x* in different lateral positions (different *x* points) where the shell structure was clearly visible in the OCT image; the mean value of the thickness over this range in *x* was used as exoskeleton thickness for that single *xy* cross-sectional image. Later, in order to measure and plot this value for the entire specimen body along the *z*-axis, the same process was repeated for a full stack of *xy* cross-sectional images collected at different *z* positions (see File S1, and Figs. S1, S2 & S3).

We also attempted to quantify the volume of internal organs of krill. To do this, as a proof of principle, we applied the following procedure: we select the OCT cross sections where the organ is visible; we manually detect the edge of the organ area for all the cross sections (*xy*-plane); the incremental volume is given by this area times the step size between one image and the following one (along the *z*-axis); the total organ volume is the integral of all the incremental volumes. If automatic measurements are needed, it may be possible to develop edge detection algorithms to identify the specific organ of interest.

A further interesting feature of our OCT system is the possibility to reconstruct 3D images of the sample. As described previously, a stack of cross-sectional *xy* images can be acquired by scanning the sample along the *z* direction. This stack is a 3D matrix of intensity data points which contains all the information to render a 3D image of the krill. Once the image is created, it can be rotated and visualized at any preferred angle using a standard imaging software such as the freely available US National Institutes of Health’s ImageJ package (imagej.net) or a home-written MATLAB code. Any cross-sectional view of interest at any angle in the sample can be easily obtained. In addition, such cross-sectional views can be used as frames to construct a movie that scans the full sample body along a particular direction of observation (See Movies S1, S2, S3 & S4).

## Results and Discussion

### 2D morphology: Anatomy thickness

**Figure 2:**
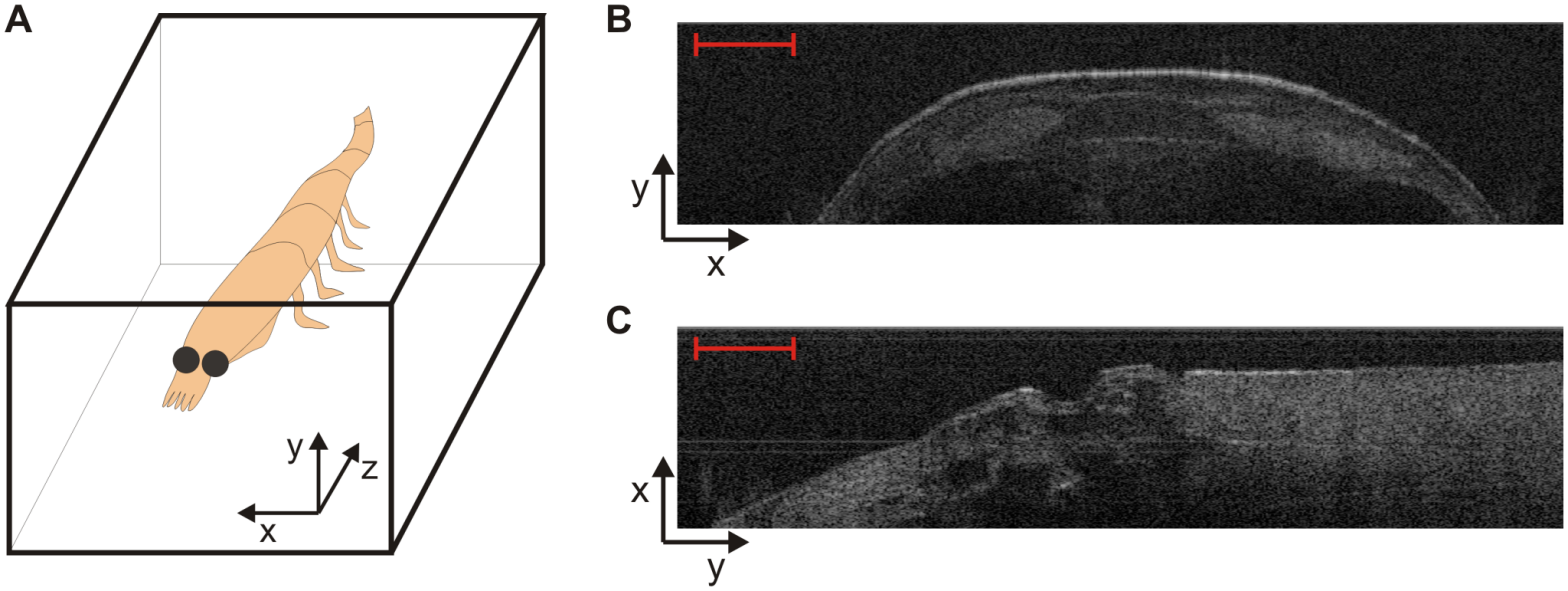
OCT images of krill carapace and pleopod joint. (A) OCT spatial reference frame axes with respect to krill position. (B) OCT cross-sectional view of krill in the carapace region; exoskeleton is clearly visible on the top. (C) OCT image of a pleopod joint. Scale bars correspond to 0.5 mm.

In Fig. 2A we provide a sketch of the spatial reference frame we used for the OCT scan of krill samples in our experiments. OCT images are available less than one second after a scan, and scans can be analysed at discrete locations on a specimen. A typical OCT cross-sectional image of the carapace region is shown in Fig. 2B; OCT is capable of resolving krill exoskeleton, on both whole animal specimens and in principle also on discarded exoskeletons. In addition, sub-surface details of internal organs are clearly visible and the internal krill morphology can be analysed for the entire sample body.

Point analysis of specimen cross-sectional images can be compared at locations that are important for particular behaviours, such as for example the potential study of pleopod joint kinematics [16]. For this purpose, an OCT image of a pleopod joint is shown in Fig. 2C.

**Figure 3:**
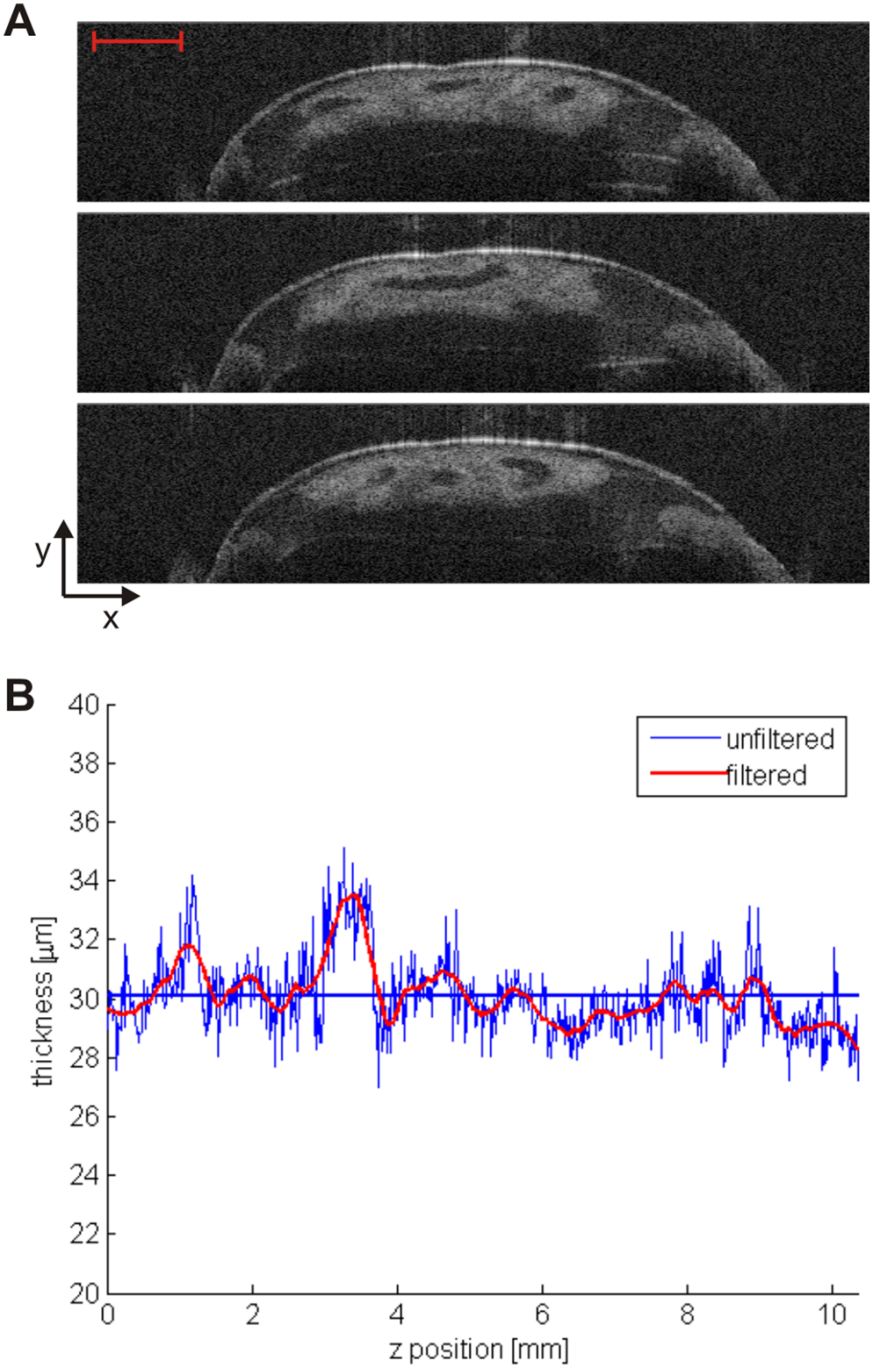
OCT data used to determine exoskeleton thickness. (A) Sequence of three OCT images for exoskeleton measurement at multiple sites; heart cross-sectional view is also clear below the krill exoskeleton. Scale bar is 0.5 mm. (B) Exoskeleton thickness profile generated from multiple OCT slices collected with 15 µm step along a 1 cm long portion of the sample body (*z*-direction); both original (blue) and filtered (red) data are shown. Average exoskeleton thickness was measured to be 30±4 µm (straight blue line).

OCT provides the capability of resolving and measuring the krill exoskeleton thickness with micrometric resolution. Multiple cross section profiles can be used to determine exoskeleton thickness and shape. As an example, Fig. 3A shows a sequence of three cross-sectional OCT images collected in three different locations along the *z*-axis from the carapace of a specimen; exoskeleton is clearly evident and thickness measurement was automatically accomplished using a custom MATLAB code (see File S1). In addition, OCT can be used to generate an exoskeleton thickness profile along a certain portion of the sample body, as shown in Fig. 3B. In this particular example, the average exoskeleton thickness was measured to be 30±4 µm along the *z*-axis on a 1-cm long portion of the krill carapace. Rapid fluctuation of the thickness value between adjacent points can be attributed to intensity noise and pixilation effects; therefore a smoothing filter (Savitzky-Golay filter, span = 50, degree = 2) was used to provide the red curve which acts as a guide to the eye. Such thickness profiles are readily amiable to intra-specimen and inter-specimen comparison where experimental living conditions want to be studied.

Initially our system was validated on commercially available prawn samples. The thicker shell of these samples enabled a direct comparison between OCT and direct mechanical measurements and a very close agreement between the two techniques was observed. The thinner exoskeleton of the krill under investigation make measurement more challenging. In the OCT image of a krill exoskeleton the top and bottom interfaces of the exoskeleton are very close to each other and in between them we observe the signal coming from scattering of material within the exoskeleton itself resulting in a single bright line feature which can be measured using the techniques described above. The structure of the krill shell is complex when viewed directly under high-resolution microscopy with multiple constituents including a cuticle that is formed of layers of individual laminae and an epidermal layer [17]. The thickness of this shell region varies depending on the position on the animal and also on the moult stage. A detailed image of a single cuticle layer from an individual sample is shown in Buchholz et al. [17] allowing the determination of the thickness of the cuticle region of approximately 10 µm at premoult stage D_0_. The value that we have obtained is larger than this so it is likely that our image is also made up of signal arising from the next cuticle forming underneath the current cuticle as well as the epidermal layer [18].

Currently, determining moult cycle stage during any physiological experiments conducted with krill can only be done retrospectively by flash freezing individual krill in liquid nitrogen, and storing them for measurement at a later date [19]. This prevents multiple observations of moult cycles on live individuals, making it difficult to account for physiological variation in individual krill and to pursue targeted experiments on particular points in the moult cycle. In principle, these results show that OCT may have the potential to help the rapid assessment of live krill moult cycle stage.

### 3D morphology: Managing the krill stock

Pre-exploitation krill population biomass is one of the parameters required to set the precautionary catch limit for managing krill stocks [20]. Typically krill biomass estimates are obtained using a fisheries independent method: hydroacoustic surveys [21]. Integral to interpreting data collected during hydroacoustic surveys is the discrimination of krill from other acoustic returns and then scaling the acoustic returns to krill density [22]. Carrying out identification and scaling requires models of target strength, a logarithmic measure of the proportion of the incident acoustic energy backscattered by the species of interest. Animal shape is an important component in target strength models [23]. Current krill target strength models that are employed to estimate krill biomass are built using cylinders to approximate the shape of krill (e.g. [24], [25]).

**Figure 4:**
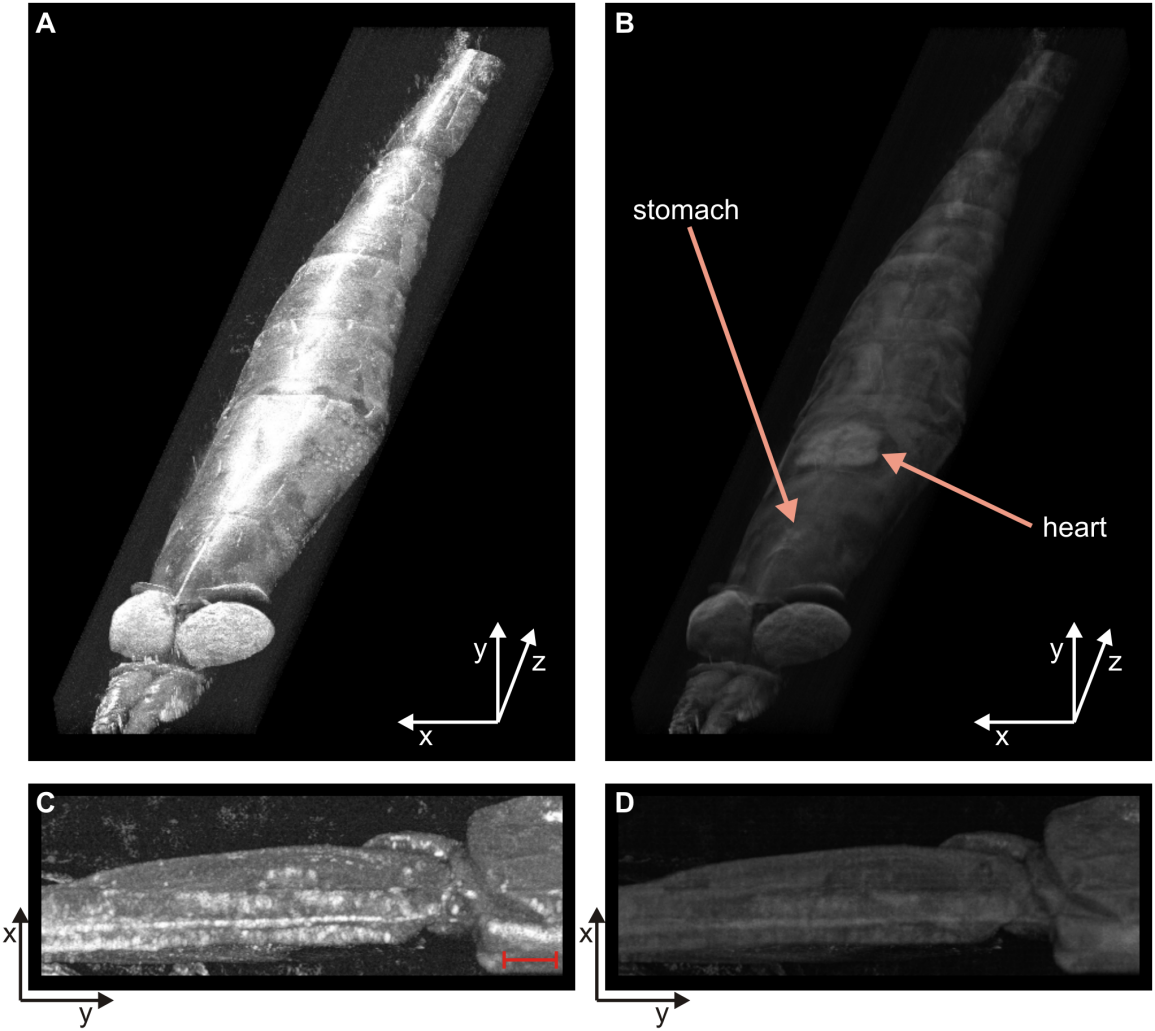
3D rendered images of krill based on multiple OCT images. Multiple OCT images have been stacked to render a 3D image of the specimen; surface (A) and sub-surface (B) features are visible depending on the rendering parameters. OCT allows the resolving of internal organs, like stomach and heart in panel B (see Figs. S1, S2 & S3 for orthogonal views; File S1 for scale, and Movies S1, S2, S3 & S4 of the full specimen). The same rendering procedure was repeated for a pleopod joint (C, D); scale bar is 0.5 mm.

For this reason, extracting krill shape from the 3D render generated with multiple OCT cross-sectional images enables the rapid creation of krill shape models. We collected a full stack of OCT images along the body of a krill specimen and reconstructed a 3D render; a volume view of the 3D image is shown in Fig. 4. The render can provide either an external view of the specimen (Fig. 4A) or a visualization in transparency to highlight sub-surface features (Fig. 4B); OCT images carry the information of internal organs and therefore the 3D image can also do the same. As an example, stomach and heart are identified in the figure.

The same process can be repeated on a smaller portion of interest. 3D render was also successfully constructed of a krill swimming appendage joint, again with two different view methods (Fig. 4C and D). As previously mentioned, swimming behaviours of krill are interesting subject of several studies.

The 3D full body image is rich in information since it allows the reconstruction of any cross-sectional view of the specimen along any preferred direction. Movies can be created to show the region of interest from different angles of view or different sections in the sample body (see Figs. S1, S2 & S3, and Movies S1, S2, S3 & S4). This represents a further powerful tool that OCT offers to the detailed study and analysis of this and other marine species of interest that are compatible with this technique.

### Area and volume measurements: Animal condition

Objective measurements of krill condition are difficult to obtain and are generally based on subjective visual observations [26]. Using the collected OCT images, we can estimate total volume of some of the internal organs, like heart, stomach, digestive gland and ovary. As an example, we measured the heart volume of a specimen; as visible from Fig. 3A and Fig. 4B, the heart was easy to locate and detect compared to other organs and heart measurements are useful for assessing stress [27] and for estimating energy requirements. For heart measurement we used 110 frames separated by 15 µm each. The estimated volume is (0.73±0.03) mm^3^; the error in the volume value is due to uncertainty on the heart edge detection and on the value of the refractive index of the tissue.

### Limitations

Clearly a limitation on OCT is the maximum depth at which images can be obtained. This depth is limited by two main factors, firstly absorption of the incident light by biological materials (e.g. blood) and secondly due to scattering which reduces both incident and backreflected light signals. Further depth penetration may be achievable by increasing the optical power incident although this may have a detrimental effect on the subject under study and/or by using wavelengths of light that are less scattered (e.g. 1300 nm). In the work described here we have used an excitation wavelength that has both a low absorption coefficient and a relatively low scattering coefficient. Similarly, we are fortunate that krill, in common with several other important animal species, are relatively transparent allowing a range of significant measurements to be undertaken. Further research in the use of advanced beam shaping techniques and in data analysis may lead to further enhancements of penetration depth permitting the application of these techniques to a greater range of studies on krill and in other species of interest.

The time taken to obtain an image is also of vital importance. In our experiments, single line scans of a particular region can be taken in a time scale that is compatible with live animal imaging although the whole body images presented would require the use of anaesthetized or euthanized samples. Subject to successful future development, other OCT methodologies, and in particular the use of optical fiber probes [28], [29] may enable single point measurements to be obtained from animals without requiring them to be removed from the aquarium. It is also of interest to note that other developments in using OCT for mapping large area capillary structures in the retina [30] have produced very high frame rates and it may, in the future, be possible to apply these techniques to living animal specimens.

Our study is based on the use of frozen samples as is common in many tissue studies. In order to examine the effect of the freezing process on the study, we have examined OCT images on samples that have been thawed and subsequently re-frozen several times. Whilst this process did show some degradation in the soft tissue structure and position, the images obtained from hard tissue (exoskeletal components) remained unchanged. The degradation observed from the thawing and re-freezing process was very similar to that which was observed by leaving the sample at room temperature for several hours. It is therefore a strong possibility that it was not the freezing process that altered the samples but the fact that the samples were degrading when not frozen. However the time taken to complete a scan was much less than the time taken for any visible degradation.

## Conclusions and Future Direction

In comparison to aquaria running costs, OCT offers a cost effective solution to sampling key metrics of small pelagic animal morphology. Being a non-invasive non-destructive technique, specimens can be sampled repeatedly or OCT can be used prior to existing sampling approaches to provide a permanent digital record.

In the future, we hope to develop an in-water fiber probe which would enable the scanning of live specimens in aquaria, with minimum disturbance offering exciting sampling opportunities. In this case, due to the lower refractive index contrast of tissue in water, OCT image quality would need to be tested to ensure high contrast and definition. This development would be particularly welcome as it opens up a raft of repeated measures statistical techniques, such as linear mixed modelling [31], potentially allowing the continuous monitoring of individual krill throughout their entire life cycle as they are exposed to various experimental conditions including projected future conditions under various Intergovernmental Panel on Climate Change (IPCC) carbon emission scenarios [3].

## Supporting Information

Figure S1 **OCT image showing the direct method for refractive index estimation.** Not-rescaled thresholded single OCT image of prawn flesh (centre) resting on a metallic plate (visible on the sides of the image). Blue line: plate position under the sample; red line: physical sample size; green line: optical path length.
(TIF)

Figure S2 **MATLAB images generated by the code for automatic measurement of exoskeleton thickness.** (A) Single raw OCT image, with exoskeleton position automatic recognition and thickness measurement in different discrete x-locations (red bold vertical bars). (B) Single intensity profile along y-axis extracted from a single x-position in the OCT image; the red bar length represents the thickness measurement at ¾ of the peak height. Numbers in the image titles show the exoskeleton thickness as FWHM of the peak intensity.
(TIF)

Figure S3 **Projection views of a krill specimen obtained from 3D OCT rendered image.** Orthogonal projections obtained from the 3D OCT render of the sample under test. Side view (yz-plane) and top view (xz-plane) are shown. Depending on the rendering parameters, (A) external shape and exoskeleton surface details or (B) sub-surface features and internal organs can be highlighted. Scale bars correspond to 2 mm.
(TIF)

File S1 **Methods, image processing MATLAB code and examples.**
(DOCX)

Movie S1 **Scanning of the front view of a krill sample.** *xy*-plane scanned along *z*; 2876 frames, 50 frames per second.
(AVI)

Movie S2 **Scanning of the side view of a krill sample.** *yz*-plane scanned along *x*; 431 frames, 20 frames per second.
(AVI)

Movie S3 **Scanning of the top view of a krill sample.** *xz*-plane scanned along *y*; 103 frames, 5 frames per second.
(AVI)

Movie S4 **360-degree rotation around the sample axis (** ***z*** **-axis).** Top view and side are visible, with both external surface details and sub-surface features clearly visible.
(AVI)
