# Supplementary figures and images for: The Application of Optical Coherence Tomography to Image Subsurface Tissue Structure of Antarctic Krill Euphausia superba
Source: PLoS One. 2014 Oct 13;9(10):e110367. doi: 10.1371/journal.pone.0110367 (PMC4195727; doi:10.1371/journal.pone.0110367)

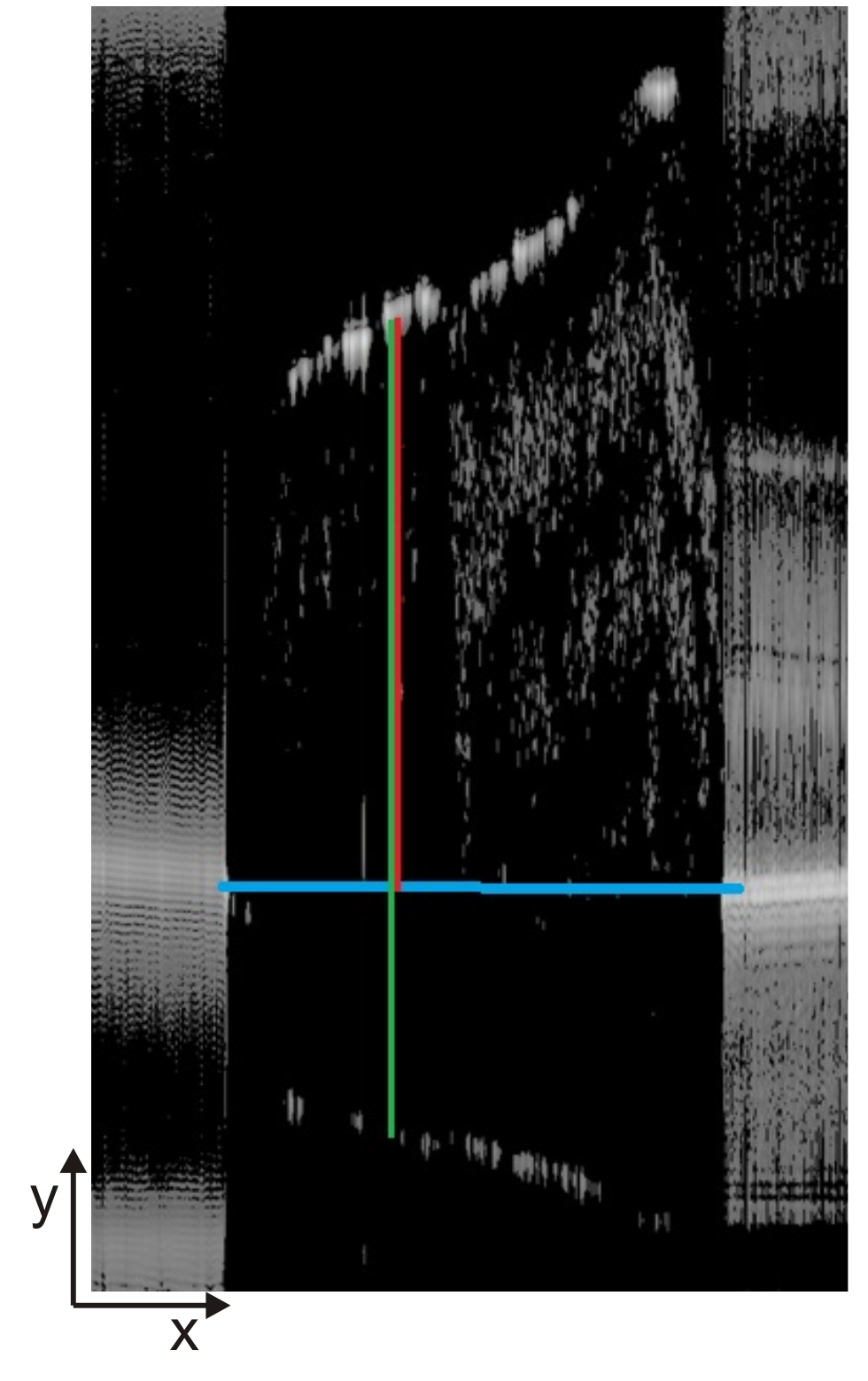

Supplement: Figure S1 — OCT image showing the direct method for refractive index estimation. Not-rescaled thresholded single OCT image of prawn flesh (centre) resting on a metallic plate (visible on the sides of the image). Blue line: plate position under the sample; red line: physical sample size; green line: optical path length. (TIF) [file pone.0110367.s001.tif]

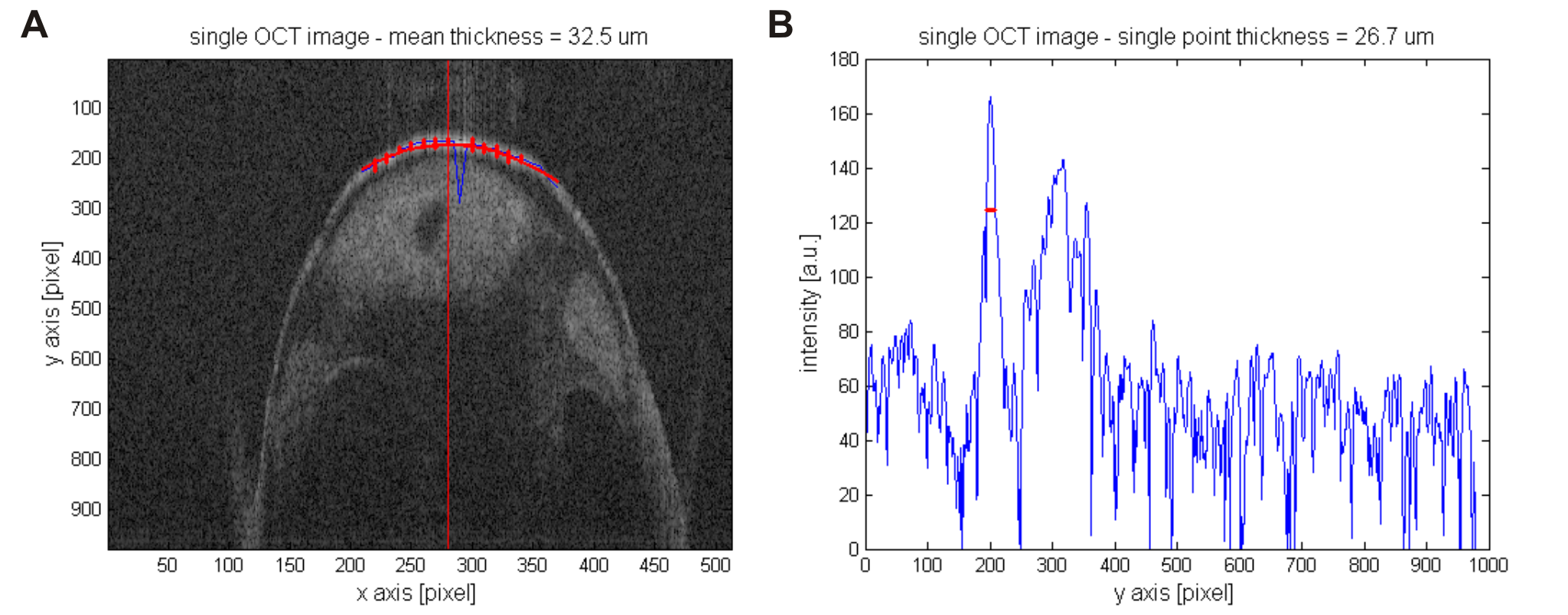

Supplement: Figure S2 — MATLAB images generated by the code for automatic measurement of exoskeleton thickness. (A) Single raw OCT image, with exoskeleton position automatic recognition and thickness measurement in different discrete x-locations (red bold vertical bars). (B) Single intensity profile along y-axis extracted from a single x-position in the OCT image; the red bar length represents the thickness measurement at ¾ of the peak height. Numbers in the image titles show the exoskeleton thickness as FWHM of the peak intensity. (TIF) [file pone.0110367.s002.tif]

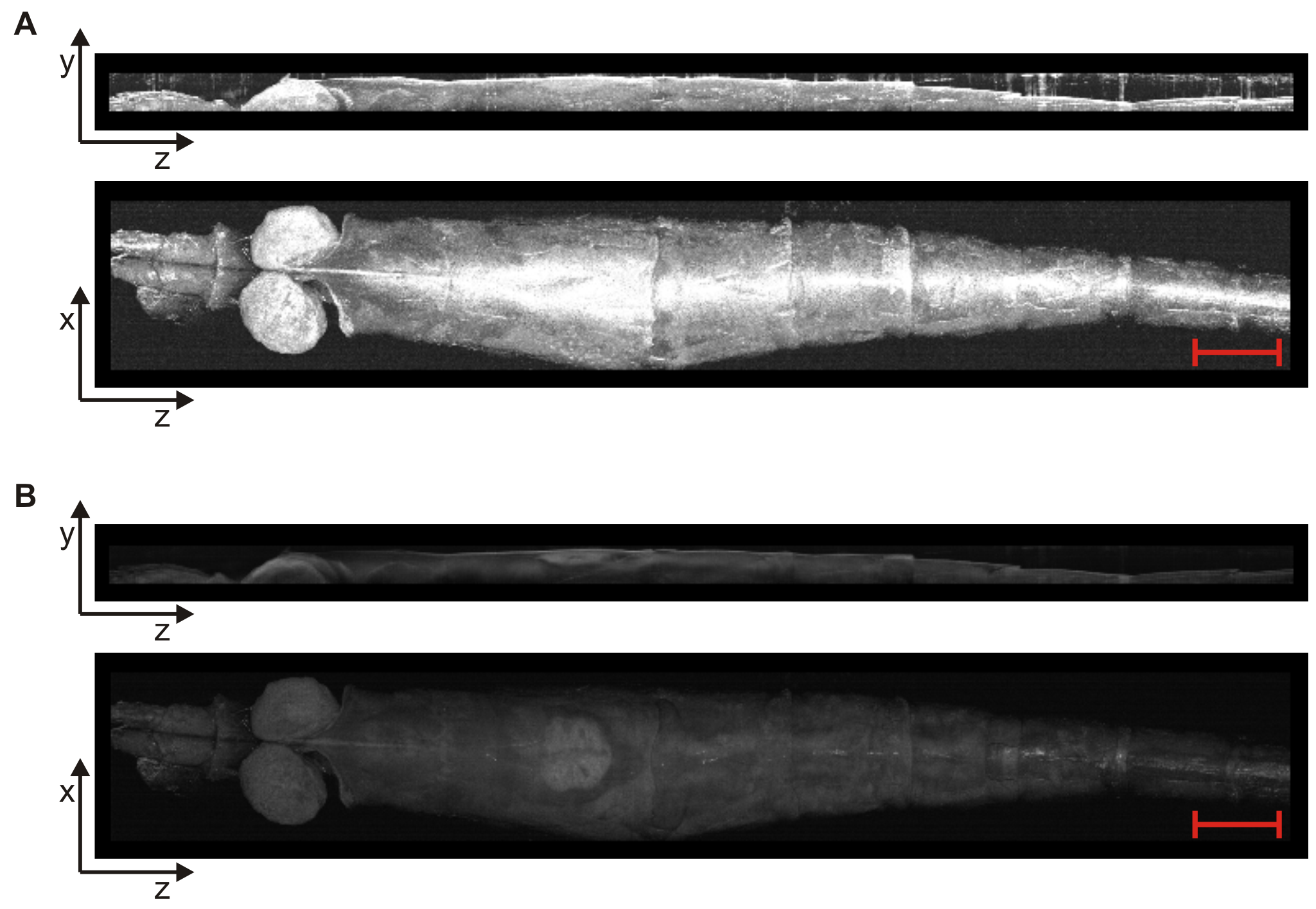

Supplement: Figure S3 — Projection views of a krill specimen obtained from 3D OCT rendered image. Orthogonal projections obtained from the 3D OCT render of the sample under test. Side view (yz-plane) and top view (xz-plane) are shown. Depending on the rendering parameters, (A) external shape and exoskeleton surface details or (B) sub-surface features and internal organs can be highlighted. Scale bars correspond to 2 mm. (TIF) [file pone.0110367.s003.tif]
