# Supplementary material for: The Application of Optical Coherence Tomography to Image Subsurface Tissue Structure of Antarctic Krill Euphausia superba
Source: PLoS One. 2014 Oct 13;9(10):e110367. doi: 10.1371/journal.pone.0110367 (PMC4195727; doi:10.1371/journal.pone.0110367)
Supplement: File S1 — Methods, image processing MATLAB code and examples. (DOCX) [file pone.0110367.s004.docx]

**Supporting Information for:**

**The application of optical coherence tomography to image subsurface tissue structure of Antarctic krill *Euphausia superba*.**

N. Bellini^1^, M.J. Cox^2^, D.J. Harper^1^, S.R. Stott^1^, P.C. Ashok^1^, K. Dholakia^1^, S. Kawaguchi^2^, R. King^2^, T. Horton^3^ and C.T.A. Brown^1,*^

1. SUPA, School of Physics and Astronomy, University of St Andrews, North Haugh, St Andrews, Fife, KY 16 9SS, U.K.

2. Australian Antarctic Division, 203 Channel Highway, Kingston, Tasmania, 7050, Australia.

3. National Oceanography Centre, University of Southampton Waterfront Campus, European Way, Southampton, SO14 3ZH, U.K.

* ctab@st-andrews.ac.uk

*Refractive index measurement of crustacean exoskeleton and flesh*

Since OCT images show the optical path rather than physical size, the refractive index value of the krill exoskeleton and flesh is important to properly rescale the vertical axis (in our case the *y*-axis) of the OCT images and obtain absolute measurements. In order to evaluate the refractive index, we used commercially available frozen raw prawn and shrimp samples, which are very similar to krill in composition and structure but bigger in size therefore easier to handle. We retrieved the refractive index using 3 different methods: i) comparison with micrometer, ii) comparison with microtome, iii) direct measurement with OCT. The first two methods require calibration of the OCT system in air: this consists in measuring the vertical range covered by the OCT image along *y*, which depends on the detection spectrometer properties. To do this, a mirror is imaged and vertically moved with a micrometric translation stage in air to scan the full visible vertical range, which in our case was measured to be 1.7 mm.

1. Comparison with micrometer. Samples with a size of roughly 100 µm were measured using a digital micrometer with 1 µm resolution providing the physical length. The same samples were then imaged and measured using OCT; this instead provides the optical length. The ratio between the optical length and the physical length was used to estimate the refractive index. This method was employed only for exoskeleton samples, since flesh samples would have been compressed by the micrometer providing misleading readings.
2. Comparison with microtome. We used a microtome to chop samples with size ranging from 400 µm to 700 µm thickness with resolution of 1 µm. The microtome cut already provides the physical length of the sample that indeed does not require other tools to be measured. Then we imaged and measured the chopped sample using OCT, which provides the optical length. Again the ratio between the two gives an estimate of the refractive index. We used this method for both exoskeleton and flesh.
3. Direct measurement with OCT. This method requires no calibration of the OCT system and no particular preparation of the sample. Figure S1 helps understanding the process: it shows an OCT image of a flesh sample placed on a horizontal partially-reflective plate (metal or glass slide); the flesh sample is placed at the centre of the image while the plate is visible on the sides. The blue line represents the approximate position of the plate under the sample. The red line represents the physical path length through the sample form the top of it to the plate. The green line represents the optical path length at same lateral position through the sample from the top to the bottom of it. The ratio between the two measurements (green/red) represents the refractive index of the sample. Multiple measurements can easily be taken from the same image at different lateral locations. This method was employed both for exoskeleton and flesh samples.

As described above, these methods were applied to exoskeleton and flesh samples and we averaged the refractive index value over several attempts to obtain a more consistent measurement. For exoskeleton samples, micrometer and microtome methods were better suited. For flesh samples instead the direct OCT method provided the most reliable refractive index values. As reported in the script, refractive index values were measured to be 1.56 ± 0.05 for exoskeleton samples and 1.45 ± 0.05 for flesh samples. Uncertainty in the exoskeleton value is mainly due to different methods used, while uncertainty in the flesh value is probably due to natural heterogeneities in the crustacean tissue.


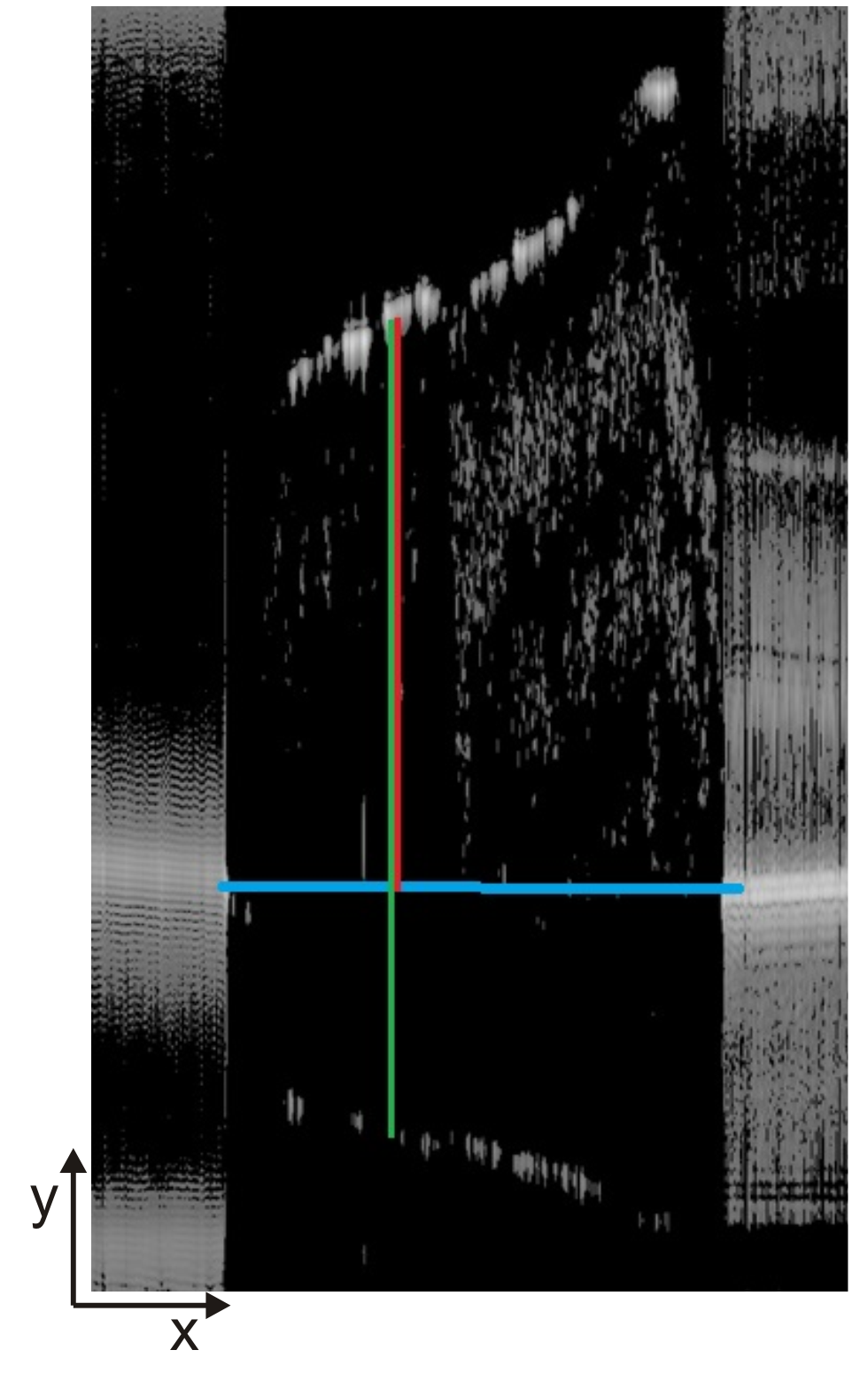


**Figure S1. OCT image showing the direct method for refractive index estimation.**

Not-rescaled thresholded single OCT image of prawn flesh (centre) resting on a metallic plate (visible on the sides of the image). Blue line: plate position under the sample; red line: physical sample size; green line: optical path length.

*Software versions*

For image processing, two software programmes were employed. We used MATLAB release R2012b from The MathWorks, Inc. (Natick, Massachusetts, USA). We also used ImageJ version 1.47 from the US National Institutes of Health (Bethesda, Maryland, USA) running with Java version 1.6.0_20 (Oracle Corporation); this software is in the public domain (http://imagej.nih.gov/ij/). Both software programmes were running under Microsoft Windows7 64-bit.

*MATLAB code for exoskeleton detection and measurement in OCT images*

The following code was written to detect and measure the krill exoskeleton thickness from a single OCT *xy*-cross-sectional image.

In this code we load the raw OCT image and then rescale measurements using the refractive index of krill exoskeleton. The method is as follows. First, the location of the top position is found. As thickness we consider the full width at half maximum (FWHM) of the exoskeleton intensity profile along *y*-axis recorded by the OCT image. To reduce the incidence of intensity noise and false edges, we directly measure the peak width at ¾ of its height; we then convert it to the FWHM considering the peak of Gaussian-like shape. In each of the single cross-sectional OCT image, thickness measurements are repeated in different *x*-positions on a range of about 1 mm. The average of these values is considered to be the exoskeleton thickness for that specific *z*-location on the specimen. False peak values are discarded from the average calculation if too far from the exoskeleton actual position.

Figure S2A shows a raw OCT image with exoskeleton automatic recognition and measurements at different discrete *x*-points along the shell. In addition, we show a single intensity profile along *y*-axis with peak width measurement at ¾ of its height (Fig. S2B).


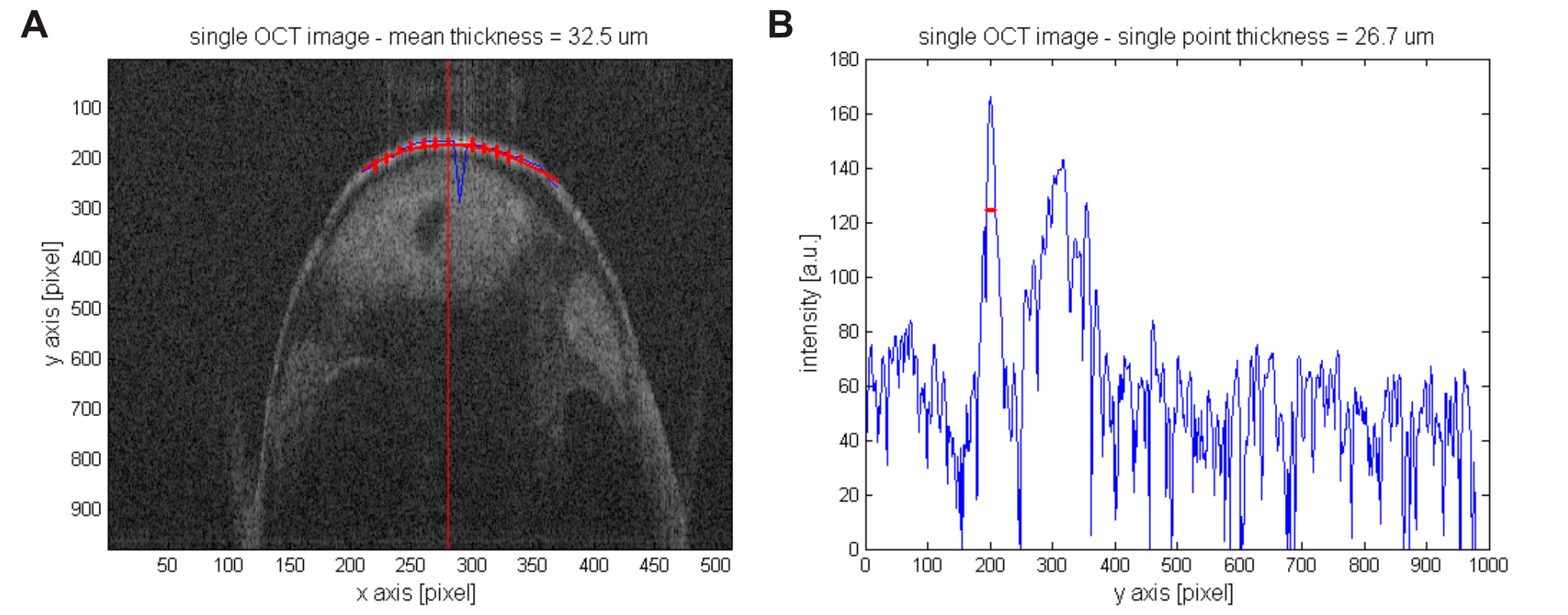


**Figure S2. MATLAB images generated by the code for automatic measurement of exoskeleton thickness.**

(A) Single raw OCT image, with exoskeleton position automatic recognition and thickness measurement in different discrete *x*-locations (red bold vertical bars). (B) Single intensity profile along *y*-axis extracted from a single *x*-position in the OCT image; the red bar length represents the thickness measurement at ¾ of the peak height. Numbers in the image titles show the exoskeleton thickness as FWHM of the peak intensity.

%measuring the shell thickness of krill

%AUTOMATIC recognition of peak position in X

%SINGLE images

%RANGE of selected X

%setting parameters

source_folder = 'data';

findxmin = 210; findxmax = 370; findxstep = 5;

xrange = 120; xstep = 10;

validity = 100;

crop_top = 45;

bg = 0; ratio = 3/4;

refr_index = 1.56; %refractive index of exoskeleton

%reading data and parameters

source_info = dir(source_folder);

source_file = fullfile(source_folder, source_info(1+2).name);

A = imread(source_file);

[ny,nx,col] = size(A);

nz = length(source_info)-2;

ny_crop = ny-crop_top;

conv = (1700/refr_index)/ny; % micrometers = conv * pixels

k = sqrt((log(1/2))/(log(3/4))); %FWHM conversion for Gaussian-like shape

A = A(crop_top+1:ny,:,:);

figure(1); image(A); hold on;

%finding the position of maximum 'posx'

A = rgb2gray(A); A = double(A);

prof = [];

for ii = findxmin:findxstep:findxmax

y = A(:,ii);

[pkx,locx] = findpeaks(y,'SORTSTR','descend');

locx = locx(1);

prof = [prof, locx];

end

x = findxmin:findxstep:findxmax;

prof_coeff = polyfit(x,prof,2);

prof_fit = polyval(prof_coeff,x);

pkx = min(prof_fit); posx = (prof_fit==pkx); posx = find(posx);

posx = findxmin + findxstep*(posx-1);

plot(x,prof,'b');

plot(x,prof_fit,'r','LineWidth',1.5);

plot(posx,1:ny_crop,'r');

%evaluate the thickness in an interval 'xrange' around 'posx'

xmin = posx-xrange/2; xmax = posx+xrange/2;

t_pos = [];

x = 1:ny_crop;

for xx = xmin:xstep:xmax

y = A(:,xx);

figure(2); plot(x,y); hold on;

[pk,loc] = findpeaks(y,'SORTSTR','descend');

pk = pk(1); loc = loc(1);

%excluding peaks too far from polynomial fit

loc_fit = polyval(prof_coeff,xx);

loc_max = loc_fit+validity/2; loc_min = loc_fit-validity/2;

if ((loc<loc_max) && (loc>loc_min))

th1=loc; th2=loc;

threshold = (pk-bg)*ratio+bg;

while ((y(th1) > threshold) && (th1 > 1))

th1 = th1-1;

end

while ((y(th2) > threshold) && (th2 < ny_crop-1))

th2 = th2+1;

end

thick = conv*(th2-th1);

thick = k*thick;

t_pos = [t_pos, thick];

figure(1); plot(xx,th1:th2,'ro','MarkerSize',1.5);

figure(2); plot(th1:th2,threshold,'ro','MarkerSize',1.5); hold off;

tit=sprintf('single OCT image - single point thickness = %0.1f um', thick);

title(tit,'fontsize',12);

end

end

%excluding MAX and MIN for calculation of mean value 't_pos'

t_pos = sort(t_pos);

t_pos_min = 2; t_pos_max = length(t_pos)-1;

t_pos = t_pos(t_pos_min:t_pos_max);

t_pos = mean(t_pos)

%labelling plots

figure(1);

tit = sprintf('single OCT image - mean thickness = %0.1f um', t_pos);

title(tit,'fontsize',12);

xlabel('x axis [pixel]','fontsize',12);

ylabel('y axis [pixel]','fontsize',12);

figure(2);

xlabel('y axis [pixel]','fontsize',12);

ylabel('intensity [a.u.]','fontsize',12);

The same basic code was used as building block for the exoskeleton thickness measurement on multiple OCT images taken along the specimen body. This code contains a loop on all the OCT images to be included. At the end of the loop, the code plots the measured exoskeleton thickness as a function of the *z*-position on the krill specimen (see Fig. 3B in the manuscript). In addition, smoothing of the curve (Savitzky-Golay filter, span = 50, degree = 2), mean value and standard deviation are calculated. The generated data set is then saved in a log file for future analysis.

*Rendering of 3D OCT images*

Another important feature of OCT technique is the capability of obtaining 3D images of the full specimen body or of a portion of it by stacking multiple OCT sections. Once the stack of OCT images is acquired, the specimens can be imaged from any angle of interest in the volume. In addition, the information of internal organs and sub-surface feature can also be made visible by adjusting transparency through the setting of suitable rendering parameters.

To do so, the stack of *xy*-rescaled OCT images of interest is imported in ImageJ where *z*-rescaling is performed. Then threshold and amplification values are adjusted to increase the image contrast. After this, we can save and store the full image stack in a single file. Using the Volume Viewer plugin of ImageJ, 3D render images are generated from the stack and rotation at any angle of view in the volume is possible. By choosing the viewing modality, we can have an external view of the sample (Max Projection mode) or a view in transparency for highlighting internal organs (Projection mode).

Showing the potential of 3D OCT imaging, Fig. S3 displays orthogonal projections of a full body of a krill specimen; while Fig. S3A shows an external view of the sample highlighting surface details, Fig. S3B shows visualization in transparency making internal organs below the krill exoskeleton easily detectable.


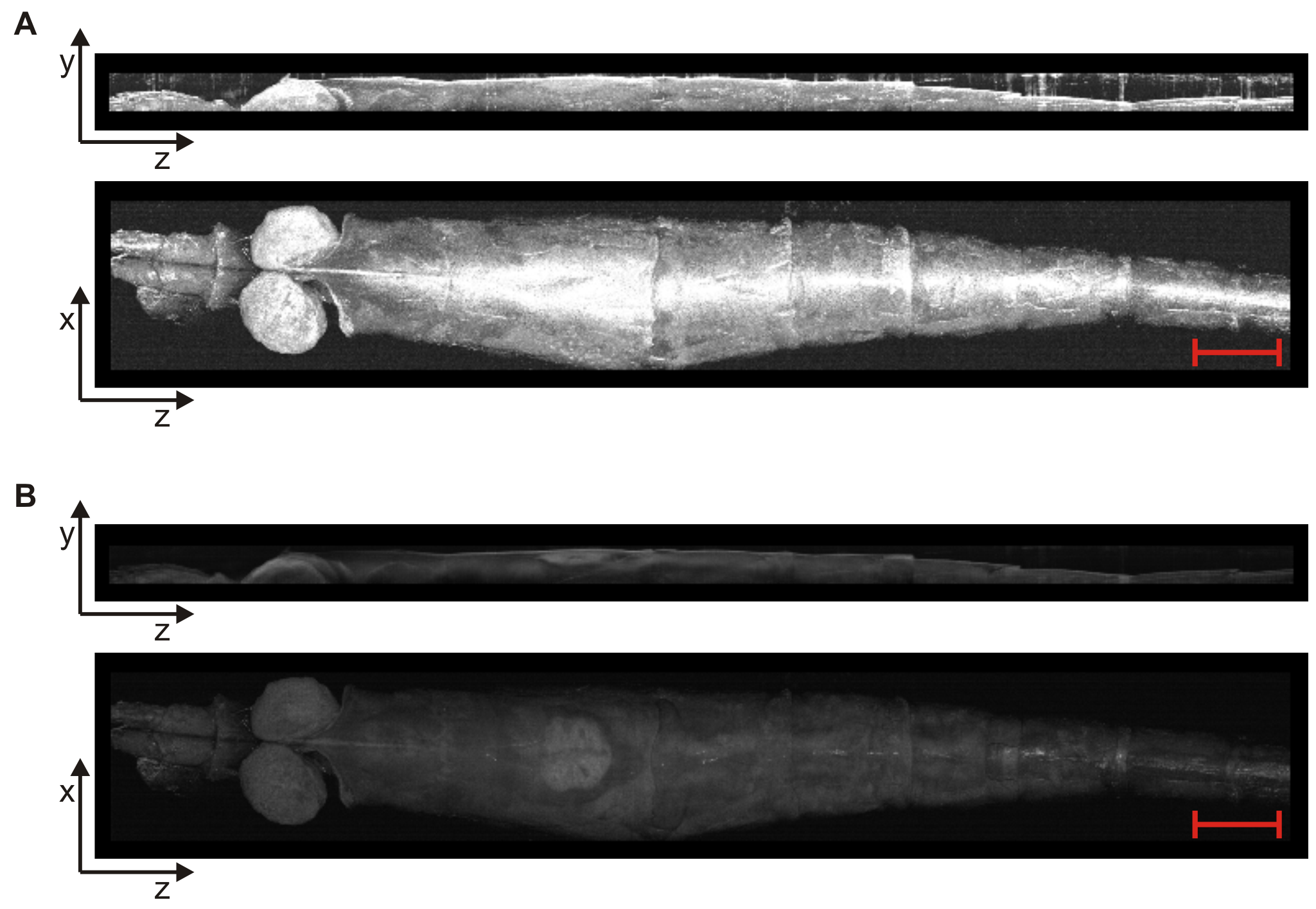


**Figure S3. Projection views of a krill specimen obtained from 3D OCT rendered image.**

Orthogonal projections obtained from the 3D OCT render of the sample under test. Side view (*yz*-plane) and top view (*xz*-plane) are shown. Depending on the rendering parameters, (A) external shape and exoskeleton surface details or (B) sub-surface features and internal organs can be highlighted. Scale bars correspond to 2 mm.

*Scan movies of full specimen body*

Using the information carried by the 3D OCT stack, scan movies can be created: it is possible to define a sectioning plane at any orientation angle in the sample volume and then scan this plane along the full body to view internal details all along the sample itself. As Supporting Information, four movies (Movies S1 to S4) can be found to demonstrate this powerful capability.

A specific home-written MATLAB code was written to load the data from the OCT image stack and generate a scan movie along a desired direction. Movies S1, S2, and S3 show scans in the front view, side view, and top view, respectively, of the full sample body. The following code reports an example in which we generate the top and side view scan movies. Movie S4 shows a rotation from the top view to the side view of the same sample, allowing both the visualization of the external exoskeleton and of some of the internal organs. This last movie (Movie S4) was generated using the 3D Viewer plugin available within ImageJ.

%creates a movie (AVI) of cross section loading all the original data set

%loading data

%saving 2 different movies with 2 different views

%setting parameters

source_folder = 'data';

source_info = dir(source_folder);

source_file = fullfile(source_folder, source_info(1+2).name);

A = imread(source_file);

[ny,nx] = size(A);

nz = length(source_info)-2;

%loading data

for ff = 1:nz

ff

source_file = fullfile(source_folder, source_info(ff+2).name);

A = imread(source_file);

P(ff,:,:) = A; %P(z,y,x)

end

%movie for XZ cross-section scan

movie_name = 'top_view.avi';

mhandle = VideoWriter(movie_name);

mhandle.FrameRate = 5;

mhandle.Quality = 100;

open(mhandle);

for ff = 1:ny

ff

for ii = 1:nx

for jj = 1:nz

% B(jj,ii) = P(jj,ff,ii); %vertical

B(ii,jj) = P(jj,ff,ii); %horizontal

end

end

writeVideo(mhandle, B);

end

close(mhandle);

%movie for YZ cross-section scan

movie_name = 'side_view.avi';

mhandle = VideoWriter(movie_name);

mhandle.FrameRate = 20;

mhandle.Quality = 100;

open(mhandle);

for ff = 1:nx

ff

% writeVideo(mhandle, P(:,:,nx-ff+1)); %vertical

writeVideo(mhandle, P(:,:,nx-ff+1)'); %horizontal

end

close(mhandle);
